# A-MPDU aggregation with optimal number of MPDUs for delay requirements in IEEE 802.11ac

**DOI:** 10.1371/journal.pone.0213888

**Published:** 2019-03-20

**Authors:** Won Hyoung Lee, Ho Young Hwang

**Affiliations:** 1 Department of Computer Engineering, Kwangwoon University, Seoul, Korea; 2 School of Computer and Information Engineering, Kwangwoon University, Seoul, Korea; Tianjin University, CHINA

## Abstract

In this paper, we propose a method that estimates an average delay of frames for each queue and finds an optimal number of aggregated Medium Access Control (MAC) Protocol Data Units (MPDUs) to maximize the system throughput with satisfying the delay requirement of each queue when using the Aggregate MPDU (A-MPDU) aggregation in IEEE 802.11ac. The delay is defined as the sum of the queuing delay and the service delay. If few frames in a queue are aggregated, the frames which remain in the queue for next transmissions may violate the target delay because of the overhead for the next transmissions such as the backoff time, Physical Layer Convergence Procedure (PLCP) preamble, and PLCP header. If many of the frames in the queue are aggregated, the frames of the queue and the other queues may violate their target delays because of a long transmission duration and a long channel occupancy. In this paper, we obtain the average delay for each queue and the optimal number of aggregated MPDUs for the delay requirement of each queue in IEEE 802.11ac. At the last, we evaluate and show the performance of our proposed method through simulations. The simulation results show that the proposed method can estimate the average delay for each queue accurately. The simulation results also show that the proposed method can obtain the violation rates on the target delays less than 0.1. Furthermore, the simulation results show that the proposed method can yield higher system throughput than other conventional methods.

## Introduction

IEEE 802.11 Distributed Coordination Function (DCF) which is based on Carrier Sense Multiple Access with Collision Avoidance (CSMA/CA) in Wireless Local Area Networks (WLANs) has been developed [1, 2]. When stations (STAs) listen to the transmissions that occur from any other STAs, the STAs pause their backoff counters until the transmissions are not sensed for the Distributed Inter-Frame Space (DIFS) duration. The IEEE 802.11 Medium Access Control (MAC) manages the retransmission of collided frames with the Binary Exponential Backoff (BEB) mechanism. Request-to-Send/Clear-to-Send (RTS/CTS) method in IEEE 802.11 DCF is used for reducing the penalty for the collision. To evaluate the throughput performance in IEEE 802.11 DCF, Bianchi [3] analyzed the system throughput in various aspects under a saturated condition with the basic and RTS/CTS methods in IEEE 802.11 DCF with a Markov chain model. Wu et al. [4] analyzed the system performance under the saturated condition with the basic and RTS/CTS methods in IEEE 802.11 DCF with considering the frame retry limit.

Since it is difficult to service the voice traffic and video traffic differentially in IEEE 802.11 DCF, IEEE 802.11e amendment has been developed. IEEE 802.11e Enhanced Distributed Channel Access (EDCA) exploits four Access Categories (ACs) according to traffics and priorities. Each AC has maximum and minimum Contention Windows (CWs), the Arbitration Inter-Frame Space (AIFS) which is used instead of the DIFS, and Transmission Opportunity (TXOP). It makes different traffics to be serviced with supporting Quality of Service (QoS). A Block Acknowledgment (BA) mechanism is introduced in IEEE 802.11e [1, 2]. In [5], the performance of IEEE 802.11e EDCA for different CWs and AIFSs according to four ACs is analyzed. Sthapit and Pyun [6] studied an implicit BA mechanism which does not transmit the BA request frame for improving the efficiency of BA mechanism in IEEE 802.11e.

IEEE 802.11n increases the bit rate up to 150 Mbps with a single spatial stream. The Multiple-Input Multiple-Output (MIMO) technology increases the bit rate up to 600 Mbps with four spatial streams. The frame aggregation in the MAC layer can reduce the overhead such as the backoff time, Physical Layer Convergence Procedure (PLCP) preamble, and PLCP header. There are three kinds of the frame aggregation as the Aggregate MAC Service Data Unit (A-MSDU), the Aggregate MAC Protocol Data Unit (A-MPDU), and the Two-level aggregation which uses both mentioned aggregations together [1, 2]. Skordoulis et al. [7] studied the MAC enhancements and analyzed the performance of the three frame aggregation schemes in IEEE 802.11n. Liu et al. [8] studied the Two-level aggregation to improve the throughput with considering overheads for the aggregation and retransmission in IEEE 802.11n.

IEEE 802.11ac is an amendment of the IEEE 802.11 standard for improving the performance of IEEE 802.11 in WLANs. It provides some features for supporting Very High Throughput (VHT). It mandatorily supports 80 MHz channel bandwidth and optionally supports 160 MHz channel bandwidth. For 160 MHz channel bandwidth, one 160 MHz channel or two discontiguous 80 MHz channels can be utilized. It also supports the 256-QAM modulation which is the Modulation and Coding Scheme (MCS) 8 or 9. The MIMO technology and the frame aggregation in IEEE 802.11n are also supported in IEEE 802.11ac. However, IEEE 802.11ac supports up to eight spatial streams while IEEE 802.11n supports up to four spatial streams. The maximum length of MPDU and the maximum length of PLCP Service Data Unit (PSDU) in IEEE 802.11ac are 11454 bytes and 1048575 bytes, respectively, while those in IEEE 802.11n are 7935 bytes and 65535 bytes [1, 2, 9].

**Fig 1:**
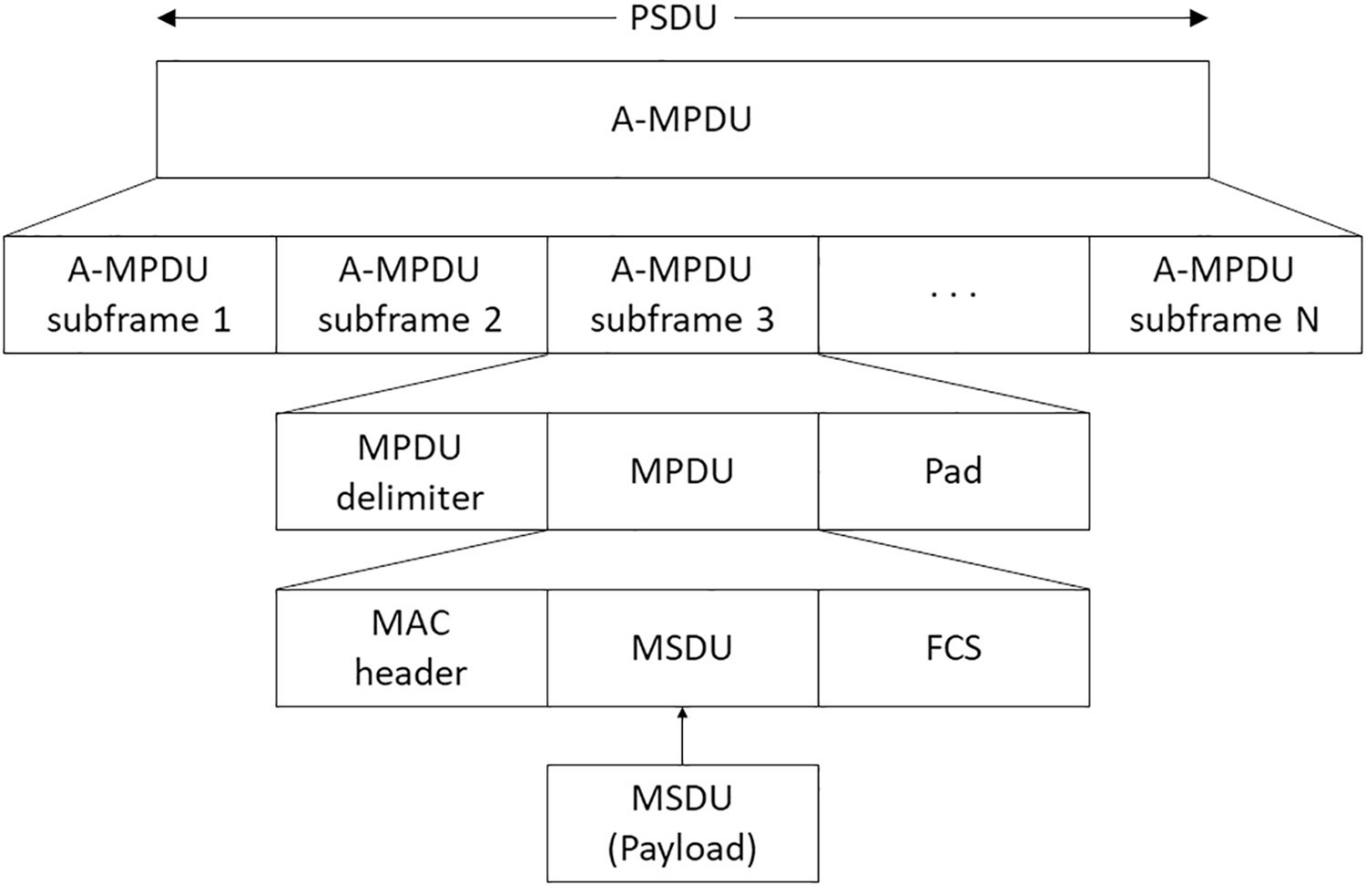
The frame format for A-MPDU [1, 9–11].

In A-MPDU aggregation, an A-MPDU consists of A-MPDU subframes and it can include up to 64 A-MPDU subframes. The frame format for A-MPDU is shown in Fig 1. To form an A-MPDU subframe, an MPDU delimiter is prepended to an MPDU and pad bits are appended to the MPDU. The pad bits are appended for making the length of A-MPDU subframe a multiple of 4 bytes. The pad bits are not appended to the last A-MPDU subframe in IEEE 802.11n while those are appended in IEEE 802.11ac. To form an MPDU, a MAC header is prepended to an MSDU and a Frame Check Sequence (FCS) is appended to the MSDU. If the VHT PLCP Protocol Data Unit (PPDU) duration is longer than the maximum VHT PPDU duration, 5.484 msec, a VHT STA cannot transmit the VHT PPDU [1, 9–11].

Our major contributions are as follows. We propose a method that estimates an average delay of MPDUs for each queue with A-MPDU aggregation and finds an optimal number of aggregated MPDUs for each queue to maximize the system throughput with satisfying the delay requirement of each queue in IEEE 802.11ac. In this paper, we define the term queuing delay of a frame as the duration from when the frame arrived at a queue to when the queue transmits the first RTS for the frame. We define the term service delay of a frame as the duration from when a queue transmits the first RTS for the frame to when the queue receives BA for the frame. We define the term delay for each queue as the sum of the queuing delay and the service delay for each queue [12–14]. Our proposed method has two steps. In the first step, the proposed method estimates the average delay of MPDUs for each queue with considering some parameters such as the number of queues, average interval between frame arrivals at each queue, the length of each queue, minimum and maximum CWs for each queue, and the target number of aggregated MPDUs for each queue in IEEE 802.11ac. To consider the finite length of each queue, we assume that newly arrived frames at the queue are dropped when the queue is full [15]. We consider that the frame arrival rate of each queue is fast enough. The proposed method can pop MPDUs from the queue to aggregate the MPDUs when transmitting the first RTS for the MPDUs. From that time, the MPDUs can wait at the extra memory until the reception of BA for the successful transmission of A-MPDU [16–18]. To estimate the average delay of MPDUs for each queue, we analyze the average interval between two consecutive successful transmissions from each queue and the average duration from when receiving BA until transmitting the first RTS for the next aggregated MPDUs from each queue in IEEE 802.11ac. In the second step, the proposed method finds the optimal number of aggregated MPDUs for each queue to maximize the system throughput with satisfying the delay requirement of each queue using the estimated average delay for each queue. If there are multiple optimal cases with ties, the proposed method selects the case with the lowest standard deviation of ratios of average delays to target delays for the queues. Through simulations, we verify that the proposed method is valid. Simulation results show that the proposed method can estimate the average delay for each queue accurately. The analytical results of the proposed method are close to simulation results when the frame arrival process follows a Poisson distribution. The simulation results also show that the proposed method can obtain the violation rates on the target delays less than 0.1. Furthermore, the simulation results show that the proposed method can yield higher system throughput than other conventional methods.

## Related works

In this section, we introduce several studies for IEEE 802.11 WLANs. Since IEEE 802.11 is the most promising mechanism in WLANs, there are many papers to improve the throughput and delay performance in IEEE 802.11 WLANs. Kim and Cho [19] studied an Adaptive TXOP Allocation (ATA) scheme for satisfying the delay bound of each packet in a queue in IEEE 802.11e EDCA. The ATA scheme checks whether each packet in a queue satisfies its delay bound or not with increasing TXOP before every transmission. The ATA scheme needs additional information about network load from an Access Point (AP) through the beacon frame. They showed the performance of the scheme through a simulation wherein an STA with multimedia traffic uses the ATA scheme while other STAs do not use TXOP. In [20], a method which finds optimal TXOP for throughput requirements under a saturated condition was studied in IEEE 802.11e EDCA. The method divided queues into two sets, one for feasible queues, another for unfeasible queues in terms of throughput requirements. The queues which satisfy their throughput requirements decrease their TXOP, and the queues which do not satisfy their throughput requirements increase their TXOP. Simulation results show that the method makes throughputs of queues close to their throughput requirements. In [19] and [20], the schemes using TXOP were studied to satisfy the throughput requirements or the delay requirements in IEEE 802.11e EDCA. In this paper, our proposed method utilizes A-MPDU aggregation instead of TXOP to satisfy the delay requirement of each queue in IEEE 802.11ac.

In [10], the performance of the A-MSDU aggregation and A-MPDU aggregation for different aggregation sizes and frame arrival rates was analyzed in IEEE 802.11n. The arrival process of frames was considered as a Poisson distribution. A Discrete Time Markov Chain (DTMC) model under an unsaturated condition was studied to have the states which describe the frame aggregation. Karmakar et al. [21] analyzed the throughput of A-MPDU aggregation in IEEE 802.11n under an unsaturated condition. They take account of A-MPDU aggregation level and data generation probability. They also analyzed MAC access delay. In this paper, our proposed method estimates the average delay for each queue which includes the MAC access delay for the queue. To estimate the average delay of MPDUs for each queue, we analyze the average interval between two consecutive successful transmissions from each queue and the average duration from when receiving BA until transmitting the first RTS for the next aggregated MPDUs from each queue in IEEE 802.11ac.

Jin et al. [22] analyzed the throughput performance with the Single-User MIMO (SU-MIMO) and the Multi-User MIMO (MU-MIMO) in uplink WLANs. Their simulation results show that if the number of STAs is small, the throughput of the SU-MIMO is higher than that of the MU-MIMO. Sharon and Alpert [9] compared the maximum throughputs of the A-MSDU aggregation, A-MPDU aggregation, and Two-level aggregation in IEEE 802.11n and IEEE 802.11ac for an error-free channel and an error-prone channel. They studied that the maximum throughput of IEEE 802.11ac is larger than or equal to that of IEEE 802.11n because of the maximum lengths of MPDU, A-MSDU, and A-MPDU. In [11], they also analyzed the performance of A-MPDU aggregation and Two-level aggregation with and without the RTS/CTS method when homogenous ACs are active in IEEE 802.11ac. They considered the transmission delay limit instead of the retransmission count limit. In their simulations, the ACs can retransmit failed frames whenever the frames are within their delay limit. They showed the maximum AppRate which is the summation of data rates for each AC with varying the number of MPDUs in PSDU and the number of ACs. In this paper, we consider the delay requirement of each queue with A-MPDU aggregation in IEEE 802.11ac. To satisfy the delay requirement of each queue, we estimate the average delay of MPDUs for each queue with considering some parameters such as the number of queues, average interval between frame arrivals at each queue, the length of each queue, minimum and maximum CWs for each queue, and the target number of aggregated MPDUs for each queue in IEEE 802.11ac.

## A-MPDU aggregation with optimal number of MPDUs for delay requirements in IEEE 802.11ac

In this section, we propose a method that estimates the average delay of MPDUs for each queue and finds the optimal number of aggregated MPDUs to maximize the system throughput with satisfying the delay requirement of each queue when the RTS/CTS method is used in IEEE 802.11ac. Our proposed method has two steps. In the first step, the proposed method estimates the average delay of MPDUs for each queue with considering some parameters such as the number of queues, average interval between frame arrivals at each queue, the length of each queue, minimum and maximum CWs for each queue, and the target number of aggregated MPDUs for each queue in IEEE 802.11ac. To consider the finite length of each queue, we assume that newly arrived frames at the queue are dropped when the queue is full [15]. We consider that the frame arrival rate of each queue is fast enough. The proposed method can pop MPDUs from the queue to aggregate the MPDUs when transmitting the first RTS for the MPDUs. From that time, the MPDUs can wait at the extra memory until the reception of BA for the successful transmission of A-MPDU [16–18]. To estimate the average delay of MPDUs for each queue, we analyze the average interval between two consecutive successful transmissions from each queue and the average duration from when receiving BA until transmitting the first RTS for the next aggregated MPDUs from each queue in IEEE 802.11ac. In the second step, the proposed method finds the optimal number of aggregated MPDUs for each queue to maximize the system throughput with satisfying the delay requirement of each queue using the estimated average delay for each queue. If there are multiple optimal cases with ties, the proposed method selects the case with the lowest standard deviation of ratios of average delays to target delays for the queues.

We consider A-MPDU aggregation of a queue with a fixed number of MPDUs for each transmission from the queue. Since we focus on the performance of A-MPDU aggregation, we assume that the queues have the same AIFS and there are *N* queues in a system [20]. The main notations are shown in Table 1. Let *T*_*w*,*i*_ denote the average waiting time for a successful transmission from the *i*-th queue, *i* = 1, 2, ⋯, *N*. Then, *T*_*w*,*i*_ can be expressed as:

$$\begin{array}{c} T_{w,i}=T_{i}-(A_{i}+O_{tx}+AIFS),i\in [1,N], \end{array}$$ (1)

where *AIFS* = *δ*⋅*AIFSN* + *SIFS* and *δ* is the length of the timeslot which is empty [1]. [*a*, *b*] is the set of integers, {*x*|*a* ≤ *x* ≤ *b*} for integers *a* and *b*. The average interval between two consecutive successful transmissions from the *i*-th queue *T*_*i*_ is defined as:

$$\begin{array}{c} T_{i}=\frac{(1-p_{b})\cdot \delta +p_{s}\cdot T_{s}+(p_{b}-p_{s})\cdot T_{c}}{p_{s,i}},i\in [1,N], \end{array}$$ (2)

where *p*_*b*_, *p*_*s*,*i*_, *p*_*s*_, *T*_*s*_, and *T*_*c*_ are defined in [20]. *p*_*b*_, *p*_*s*,*i*_, and *p*_*s*_ are the probability that the status of the channel is sensed as busy, the probability that a successful transmission from the *i*-th queue occurs, and the probability that a successful transmission occurs in the channel, respectively. *T*_*s*_ and *T*_*c*_ are the average times that a successful transmission and a collision occur, respectively, in the channel. Let *T*_*service*_, *T*_*payload*_, and *T*_*tail*_ be the transmission durations for PLCP service field, payload, and PLCP tail field, respectively. Then, the duration for transmission of A-MPDU from the *i*-th queue, *A*_*i*_ can be expressed as:

$$\begin{array}{c} A_{i}=T_{service}+F_{i}\cdot (T_{payload}+O_{A-MPDU})+T_{tail},i\in [1,N], \end{array}$$ (3)

where *F*_*i*_ is the target number of aggregated MPDUs for the *i*-th queue, *O*_*A*−*MPDU*_ is A-MPDU overhead time for MPDU delimiter, MAC header, FCS, and pad bits. In Eq (1), the overhead time for a successful transmission *O*_*tx*_ can be expressed as:

$$\begin{array}{c} O_{tx}=T_{RTS}+3\cdot SIFS+T_{CTS}+T_{preamble}+T_{BA}, \end{array}$$ (4)

where *T*_*RTS*_, *T*_*CTS*_, *T*_*preamble*_, and *T*_*BA*_ are the transmission durations for RTS, CTS, PLCP preamble, and BA, respectively.

**Table 1:** Notations.

Notation	Meaning
*N*	The number of queues
*p*_*b*_	The probability that the status of the channel is sensed as busy
*p*_*s*,*i*_	The probability that a successful transmission from the *i*-th queue occurs
*p*_*s*_	The probability that a successful transmission occurs in the channel
*p*_*b*,\*i*_	The probability that the status of the channel is sensed as busy during the backoff procedure of the *i*-th queue
*p*_*s*,\*i*_	The probability that a successful transmission occurs in the channel during the backoff procedure of the *i*-th queue
*A*_*i*_	The duration for transmission of A-MPDU from the *i*-th queue
*F*_*i*_	The target number of aggregated MPDUs for the *i*-th queue
*I*_*i*_	The average interval between frame arrivals at the *i*-th queue
*D*_*i*_	The average delay for the *i*-th queue
*D*_*ij*_	The average delay of the *j*-th frame among *F*_*i*_ frames for the *i*-th queue
*T*_*w*,*i*_	The average waiting time for a successful transmission from the *i*-th queue
*T*_*i*_	The average interval between two consecutive successful transmissions from the *i*-th queue
*T*_*s*_	The average times that a successful transmission occurs in the channel
*T*_*c*_	The average times that a collision occurs in the channel
*T*_*service*_	The transmission duration of PLCP service field
*T*_*payload*_	The transmission duration of payload
*T*_*tail*_	The transmission duration of PLCP tail field
*T*_*RTS*_	The transmission duration of RTS
*T*_*CTS*_	The transmission duration of CTS
*T*_*preamble*_	The transmission duration of PLCP preamble
*T*_*BA*_	The transmission duration of BA
*δ*	The length of the timeslot which is empty
*T*_*slot*,*i*_	The average duration of a virtual slot during the backoff procedure of the *i*-th queue.
*T*_*BO*,*i*_	The average duration from when receiving BA until transmitting the first RTS for the next aggregated MPDUs from the *i*-th queue
*W*_*i*_	The minimum CW of the *i*-th queue
*L*_*i*_	The length of the *i*-th queue
*O*_*tx*_	The overhead time for a successful transmission
*O*_*A*−*MPDU*_	A-MPDU overhead time for MPDU delimiter, MAC header, FCS, and pad bits

**Fig 2:**
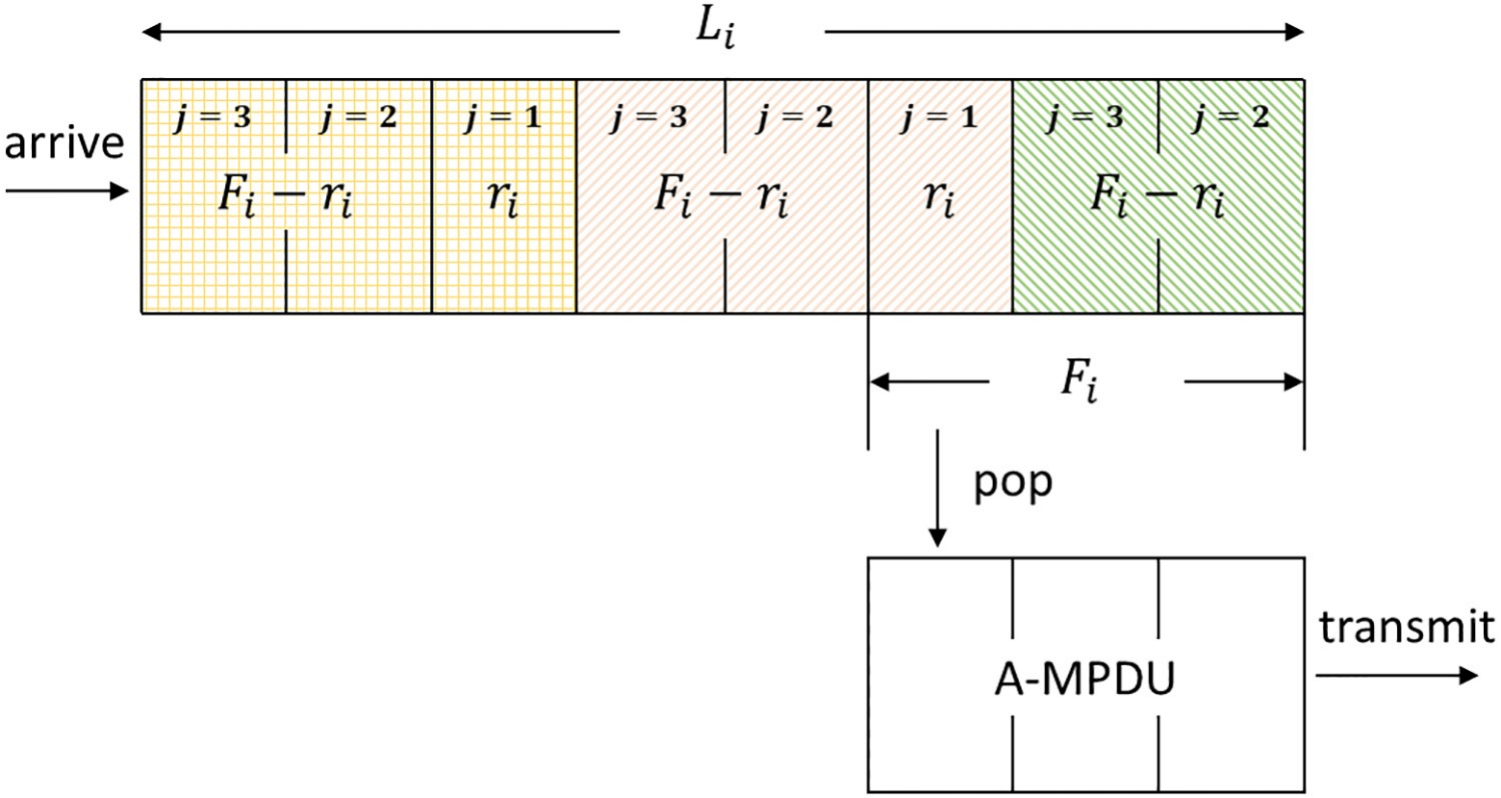
Example of a status for a queue [16–18].

We assume that newly arrived frames at a queue are dropped when the queue is full [15]. Let *I*_*i*_ denote the average interval between frame arrivals at the *i*-th queue. We assume that each frame arrives with the same interval *I*_*i*_ like Constant Bit Rate (CBR) traffic [23]. Let *D*_*i*_ denote the average delay for the *i*-th queue. Then, *D*_*i*_ can be expressed as:

$$\begin{array}{c} D_{i}\;≃\;\sum_{j=1}^{F_{i}}\frac{D_{ij}}{F_{i}},i\in [1,N], \end{array}$$ (5)

where *D*_*ij*_ is the average delay of the *j*-th frame among *F*_*i*_ frames for the *i*-th queue. The proposed method can pop *F*_*i*_ MPDUs from the queue to aggregate the MPDUs when transmitting the first RTS for the MPDUs as shown in Fig 2. From that time, the MPDUs can wait at the extra memory until the reception of BA for the successful transmission of A-MPDU [16–18]. Then, *D*_*ij*_ can be obtained as:

$$\begin{array}{c} D_{i,j}\;≃\;\{\begin{array}{c} T_{i}\cdot (Q_{i}-1)+T_{w,i}+O_{tx}+A_{i}-T_{BO,i}-I_{i}\cdot (j-\frac{1}{2}),j\in [1,r_{i}]\\ T_{i}\cdot Q_{i}+T_{w,i}+O_{tx}+A_{i}-T_{BO,i}-I_{i}\cdot (j-\frac{1}{2}),j\in [r_{i}+1,F_{i}] \end{array},i\in [1,N], \end{array}$$ (6)

where *T*_*BO*,*i*_, *T*_*slot*,*i*_, *Q*_*i*_, and *r*_*i*_ can be expressed as:

$$T_{BO,i}=\frac{W_{i}-1}{2}\cdot T_{slot,i},i\in [1,N],$$ (7)

$$T_{slot,i}=(1-p_{b,\backslash i})\cdot \delta +p_{s,\backslash i}\cdot T_{s}+(p_{b,\backslash i}-p_{s,\backslash i})\cdot T_{c},i\in [1,N],$$ (8)

$$Q_{i}=\lfloor \frac{L_{i}}{F_{i}}\rfloor +1,i\in [1,N],$$ (9)

$$r_{i}=F_{i}-(L_{i}\;\text{mod}\;F_{i}),i\in [1,N],$$ (10)

where ⌊*n*⌋ is the largest integer smaller than or equal to real number *n*. (*a* mod *b*) is the remainder of *a* divided by *b*. *p*_*b*,\*i*_ and *p*_*s*,\*i*_ are the probability that the status of the channel is sensed as busy and the probability that a successful transmission occurs in the channel, respectively, during the backoff procedure of the *i*-th queue [24]. *T*_*slot*,*i*_ is the average duration of a virtual slot [5] during the backoff procedure of the *i*-th queue. To obtain *T*_*w*,*i*_, *T*_*i*_, and *T*_*slot*,*i*_, we use Wu’s model [4]. Some parameters such as the minimum and maximum CWs of the *i*-th queue affect *T*_*w*,*i*_, *T*_*i*_, and *T*_*slot*,*i*_. *T*_*BO*,*i*_ is the average duration from when receiving BA until transmitting the first RTS for the next aggregated MPDUs from the *i*-th queue. *W*_*i*_ is the minimum CW of the *i*-th queue. *L*_*i*_ is the length of the *i*-th queue, i.e., the maximum number of frames which can be queued at the *i*-th queue.

Fig 2 shows an example of a status for a queue where the length of the queue is 8 and the target number of aggregated MPDUs is 3. That is, *L*_*i*_ = 8 and *F*_*i*_ = 3, so *r*_*i*_ = 3 − (8 mod 3) = 1. In Fig 2, for the three frames which are aggregated and transmitted, let’s assume that the first two frames have arrived after the *g*-th successful transmission, then the last one frame has arrived after the (*g* + 1)-th successful transmission. Thus, we can estimate the average delay for the *i*-th queue by dividing *F*_*i*_ frames into two sets which consist of *F*_*i*_ − *r*_*i*_ frames and *r*_*i*_ frames, respectively. In this paper, we consider that the frame arrival rate 1/*I*_*i*_ is fast enough. Otherwise, the actual average delay for the *i*-th queue may be less than *D*_*i*_ since Eqs (1), (2), and (7) to (10) are obtained under a saturated condition.

Now, we find the optimal number of aggregated MPDUs for each queue to maximize the system throughput with satisfying the delay requirement of each queue in IEEE 802.11ac. The proposed method estimates the average delay of each queue to satisfy the delay requirement of each queue. The average delay of each MPDU *D*_*ij*_ depends on the target number of aggregated MPDUs *F*_*i*_ and the length of each queue *L*_*i*_. Thus, we propose a method to find an optimal number of aggregated MPDUs for each queue $F_{i}^{*}$ to satisfy the delay requirement of each queue when the frame arrival interval *I*_*i*_ and the length of each queue *L*_*i*_ are given. It is possible to simply guess that the average delay of each MPDU for the *i*-th queue should be less than the target delay of the *i*-th queue *d*_*i*_. Then, we can obtain the constraint (11) with the number of aggregated MPDUs for each queue ***F*** = (*F*_1_, *F*_2_, ⋯, *F*_*N*_) as:

$$\begin{array}{c} D_{ij}(F)\leq d_{i},j\in [1,F_{i}],i\in [1,N]. \end{array}$$ (11)

However, if *D*_*ij*_ is close to *d*_*i*_, a half of the transmitted MPDUs may violate the target delay of the *i*-th queue since *D*_*ij*_ is an average value. Thus, we use the constraint (12) instead of the constraint (11) with considering the weight value *ω*_*i*_ which is the real value (0 < *ω*_*i*_ ≤ 1). The constraint (12) means that the average delay of the *i*-th queue should be less than or equal to the weighted target delay of the *i*-th queue as:

$$\begin{array}{c} D_{i}(F)\leq \omega_{i}\cdot d_{i},i\in [1,N]. \end{array}$$ (12)

Moreover, to maximize the system throughput with satisfying the constraint (12), we use Eq (13) as:

$$F_{th}=\underset{F}{\mathrm{argmax}}\sum_{i=1}^{N}F_{i}.$$ (13)

The large number of aggregated MPDUs for each queue can cause a high system throughput since we consider the RTS/CTS method. If there is more than one value ***F***_*th*_ with ties from Eqs (13) and (14) is used to select one of them as:

$$\begin{array}{c} F^{*}=\underset{F_{th}}{\mathrm{argmin}}\text{std}(D_{norm}), \end{array}$$ (14)

where ***D***_*norm*_ = (*D*_1_/*d*_1_, *D*_2_/*d*_2_, ⋯, *D*_*N*_/*d*_*N*_) and std (***x***) is the standard deviation of elements of vector ***x***. Through Eqs (13) and (14), we can obtain $F^{*}=(F_{1}^{*},F_{2}^{*},\cdots ,F_{N}^{*})$ by considering not only the summation of elements of vector ***F*** but also the standard deviation of ratios of the average delays to the target delays for the queues ***D***_*norm*_.

## Simulation results

In this section, we evaluate the performance of our proposed method through simulations. First, we evaluate the performance of our proposed method in terms of the average delay with A-MPDU aggregation. Then, we evaluate the performance of the proposed method in terms of the violation rate on the target delay. We use Network Simulator 3 (NS-3) version 26 for simulations [18]. The input parameters for simulations are shown in Table 2 [1, 18]. In NS-3, when the size of A-MPDU is smaller than the maximum A-MPDU size, the MPDUs for a queue can be aggregated up to 64 MPDUs which is given as the maximum number of aggregated MPDUs in NS-3 simulations. We set the maximum A-MPDU size in NS-3 enough to aggregate the MPDUs as the target number of aggregated MPDUs. In NS-3, each queue may aggregate the MPDUs less than the target number of aggregated MPDUs whenever it has less MPDUs than the target number of aggregated MPDUs. This may cause the difference between the analytical results and the simulation results. We consider that each STA has a queue with best effort traffic. We run NS-3 simulations 10 times for each point of each figure in this section and we show the average and the standard deviation of the simulation results in each figure.

**Table 2:** Input parameters for simulations [1, 18].

Input parameter set I	
**Parameter**	**Value**
MPDU size	502 bytes
Data rate	54 Mbps
Control rate	13.5 Mbps
The length of each queue	100
Input parameter set II	
**Parameter**	**Value**
MPDU size	1498 bytes
Data rate	180 Mbps
Control rate	13.5 Mbps
The number of STAs *N*	9
The number of class *C*	3
STA indices for *class*[*c*], *c* ∈ [1, *C*]	{3*c* − 2, 3*c* − 1, 3*c*}
The length of each queue	150
Input parameter set III	
**Parameter**	**Value**
MPDU size	1498 bytes
Data rate	54 Mbps
Control rate	13.5 Mbps
The number of STAs *N*	9
The number of class *C*	3
STA indices for *class*[*c*], *c* ∈ [1, *C*]	{3*c* − 2, 3*c* − 1, 3*c*}
The length of each queue	100
Common parameters	
**Parameter**	**Value**
RTS size	20 bytes
CTS size	14 bytes
BA size	32 bytes
MPDU delimiter size	4 bytes
MAC header size	26 bytes
FCS size	32 bits
PSDU service field size	16 bits
PSDU tail field size	6 bits
The minimum CW	15
The maxmimum CW	1023
AIFSN	3
Slot time	9 *μ*sec
SIFS time	16 *μ*sec
PLCP preamble time	40 *μ*sec
RTS transmission duration	36 *μ*sec
CTS transmission duration	44 *μ*sec
BA transmission duration	32 *μ*sec
CTS timeout	75 *μ*sec

**Fig 3:**
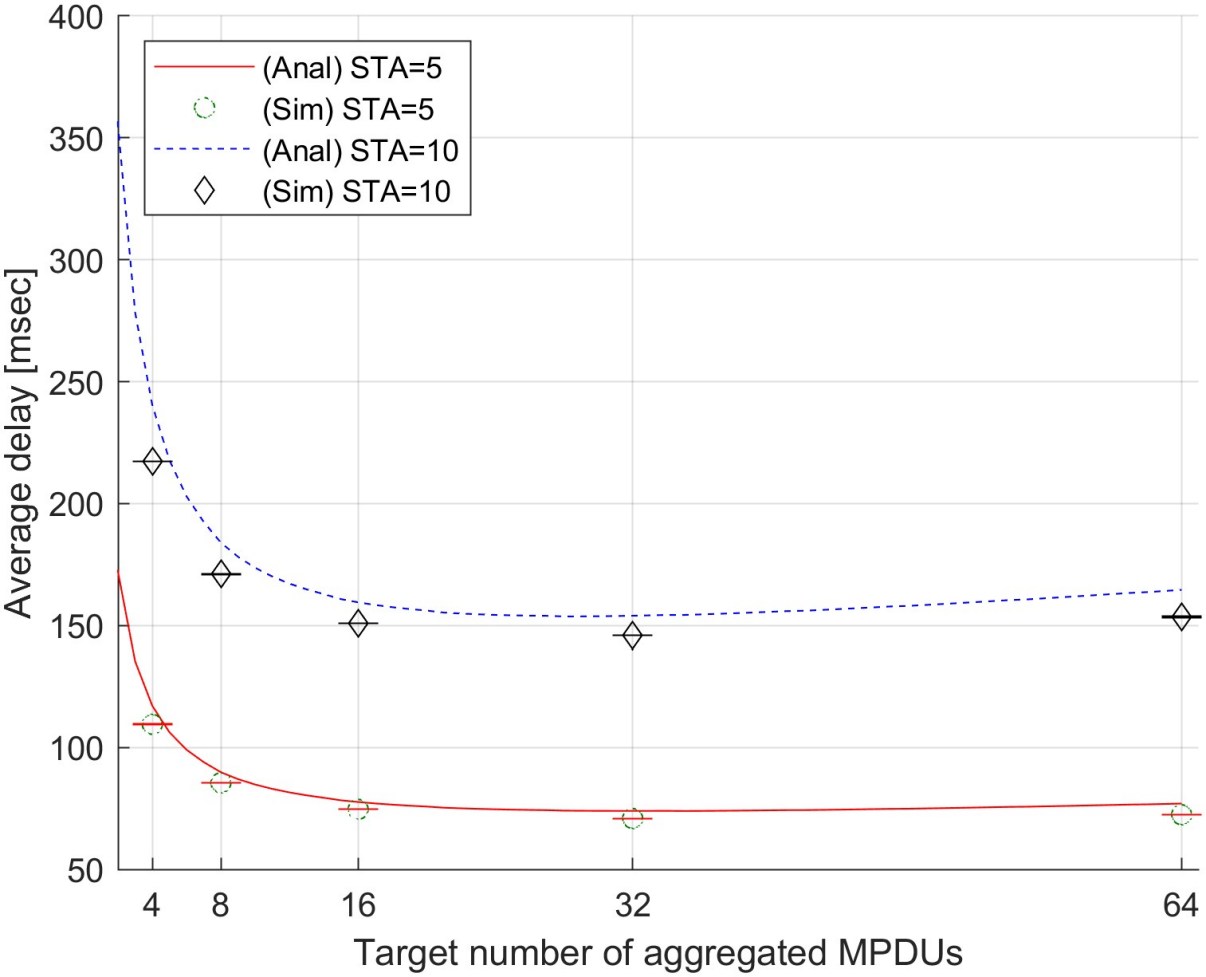
Average delay with varying the target number of aggregated MPDUs.

Fig 3 shows the average delay with varying the target number of aggregated MPDUs. To validate the proposed model, we run the simulation in NS-3 with input parameter set I when the numbers of STAs are 5 and 10, respectively. To compare the analytical results with the simulation results, we vary the target number of aggregated MPDUs as 4, 8, 16, 32, and 64. In Fig 3, the analytical results are shown as lines and the averages of the simulation results are shown as symbols. The error bar on each symbol indicates the standard deviation of the simulation results. When the number of STAs is 5, the analytical results are 117.09, 89.85, 77.64, 73.99, and 77.00 msec, while the simulation results are 109.5, 85.53, 74.63, 70.88, and 72.42 msec. When the number of STAs is 10, the analytical results are 240.39, 183.98, 159.52, 154.03 and 164.64 msec, while the simulation results are 217.27, 171.10, 150.93, 146.01, and 153.54 msec. Through the NS-3 simulations, the results show that the proposed method can estimate the average delay accurately.

Let $N_{i}^{violated}$, $N_{i}^{total}$, *R*_*c*_, and *R*_*avg*_ denote the number of MPDUs which violate the target delay of the *i*-th STA, the number of transmitted MPDUs from the *i*-th STA, the violation rate on the target delay of each class *c* ∈ [1, *C*], and the average violation rate on the target delays, respectively. Then, *R*_*c*_ and *R*_*avg*_ can be obtained as:

$$R_{c}=\frac{\sum_{i\in class[c]}N_{i}^{violated}}{\sum_{i\in class[c]}N_{i}^{total}},c\in [1,C],$$ (15)

$$R_{avg}=\frac{\sum_{i=1}^{N}N_{i}^{violated}}{\sum_{i=1}^{N}N_{i}^{total}}.$$ (16)

**Fig 4:**
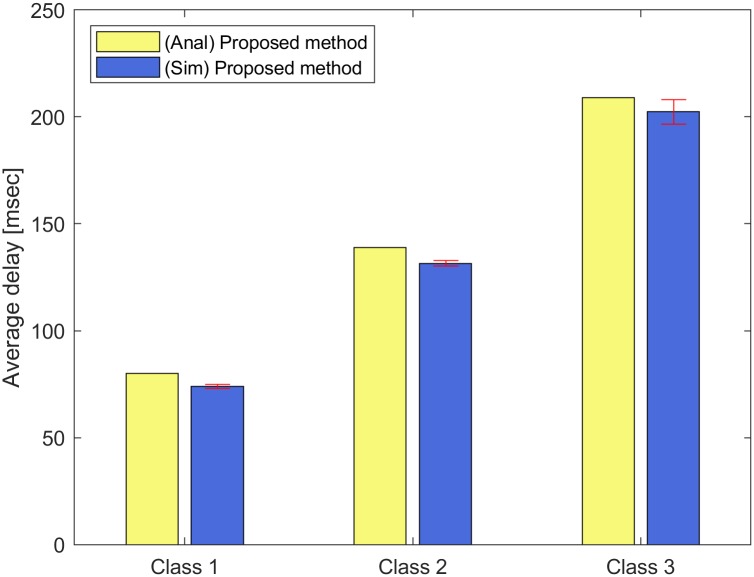
Average delay of each class.

**Fig 5:**
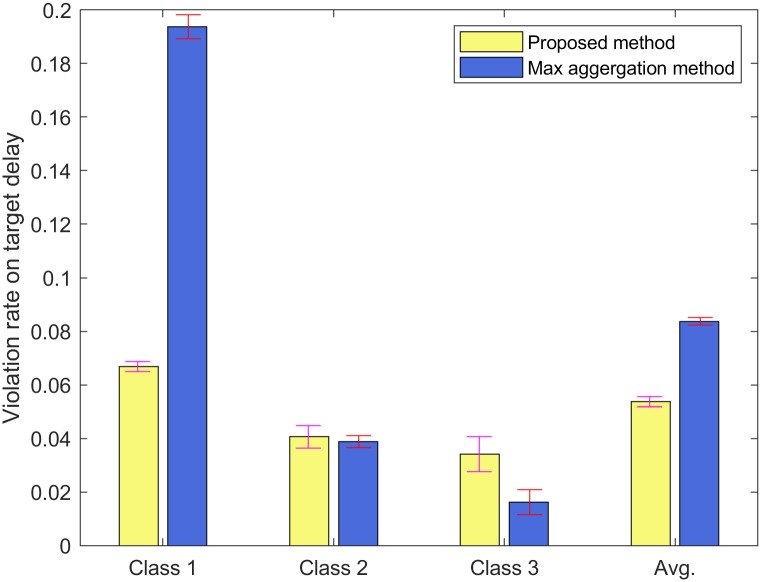
The violation rate on the target delay of each class.

For Figs 4 and 5, we run NS-3 simulations to show the violation rates on the target delays with input parameter set II. The error bar indicates the standard deviation of the simulation results. We consider three classes (*C* = 3) and each class consists of three STAs (*N* = 9). These classes have different delay requirements and frame arrival intervals. The target delay of each class *c* ∈ [1, 3] is given by the vector (160, 320, 480) msec, and the frame arrival interval of each class *c* ∈ [1, 3] is given by the vector (100, 200, 300) *μ*sec. From Eqs (1) to (14), we can obtain the target numbers of aggregated MPDUs for each class *c* ∈ [1, 3] as 64, 33, and 21, respectively, with the weight value *ω*_*i*_ = 0.5, *i* ∈ [1, 9] in the constraint (12). Fig 4 shows the average delay of each class *c* ∈ [1, 3]. In Fig 4, the analytical results for the average delays of classes *c* ∈ [1, 3] are 79.93, 139.00, and 208.97 msec, while the simulation results for those are 73.98, 131.47, and 202.35 msec. The simulation results are little smaller than the analytical results, but they are close.

Fig 5 shows the violation rate on the target delay of each class *c* ∈ [1, 3] and the average violation rate on the target delays. In Fig 5, we also simulate the max aggregation method which aggregates the MPDUs for each queue up to 64 MPDUs to compare the performance of the proposed method with that of the max aggregation method. In Fig 5, the violation rates on the target delays of classes *c* ∈ [1, 3] for the proposed method are 0.067, 0.041, and 0.034, while those for the max aggregation method are 0.194, 0.039, and 0.016. For class 1, the violation rate on the target delay for the max aggregation method is approximately three times higher than that for the proposed method. Since the target numbers of aggregated MPDUs for classes 2 and 3 of the proposed method are less than those of the max aggregation method, the proposed method can aggregate less MPDUs for classes 2 and 3 than the max aggregation method. It means that the average duration of a virtual slot during the backoff procedure for class 1 of the proposed method can be shorter than that of the max aggregation method. Thus, the average delay for class 1 of the proposed method as 73.98 msec is shorter than that of the max aggregation method as 116.34 msec with input parameter set II. Due to this reason, the violation rate on the target delay for class 1 of the proposed method is lower than that of the max aggregation method in Fig 5. On the other hand, class 3 of the proposed method may need more successful transmissions than class 3 of the max aggregation method in order to transmit the same number of MPDUs successfully due to less target number of aggregated MPDUs. Thus, the average delay for class 3 of the proposed method as 202.35 msec is longer than that of the max aggregation method as 109.22 msec with input parameter set II. Due to this reason, the violation rate on the target delay for class 3 of the proposed method is higher than that of the max aggregation method in Fig 5.

The results show that the class which has shorter target delay has a larger violation rate on the target delay. The average violation rates on the target delays for the proposed method and the max aggregation method are 0.054 and 0.084, respectively. The results show that the average violation rate on the target delays of the max aggregation method is higher than that for the proposed method.

**Fig 6:**
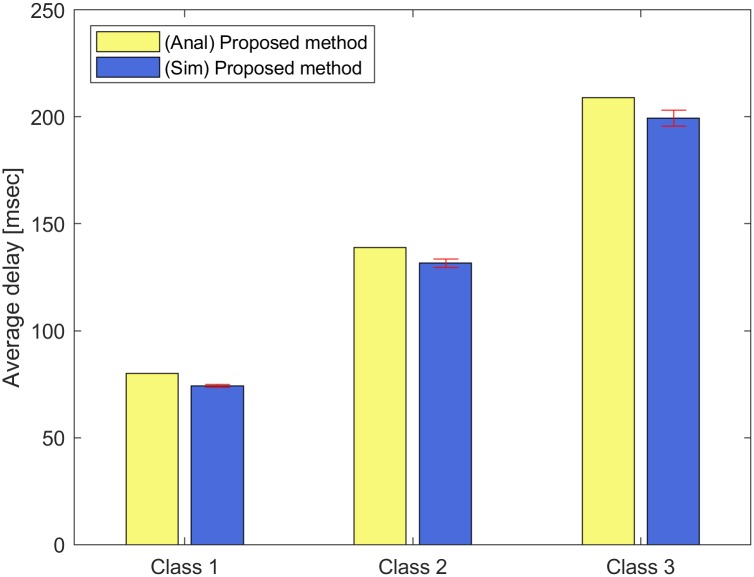
Average delay of each class under a Poisson distribution.

**Fig 7:**
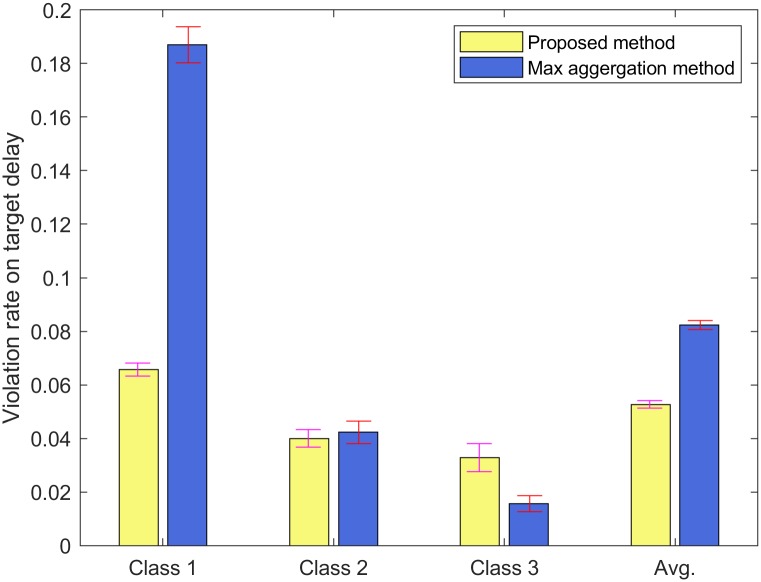
The violation rate on the target delay of each class under a Poisson distribution.

For Figs 6 and 7, we run NS-3 simulations when the frame arrival process follows a Poisson distribution. We also use the input parameter set II. The error bar indicates the standard deviation of the simulation results. Fig 6 shows the average delay of each class *c* ∈ [1, 3]. In Fig 6, the analytical results for the average delays of classes *c* ∈ [1, 3] are 79.93, 139.00, and 208.97 msec, while the simulation results for those are 74.22, 131.60, and 199.51 msec. Even though the frame arrival process follows a Poisson distribution, the analytical results of the proposed method are close to simulation results. Fig 7 shows the violation rate on the target delay of each class *c* ∈ [1, 3] when the frame arrival process follows a Poisson distribution. In Fig 7, the violation rates on the target delays of classes *c* ∈ [1, 3] for the proposed method are 0.066, 0.040, and 0.033, while those for the max aggregation method are 0.187, 0.042, and 0.016. The average violation rates on the target delays for the proposed method and the max aggregation method are 0.053 and 0.082, respectively. The results show that the proposed method can obtain the violation rates on the target delays less than 0.1 while the max aggregation method may not when the frame arrival process follows a Poisson distribution.

**Fig 8:**
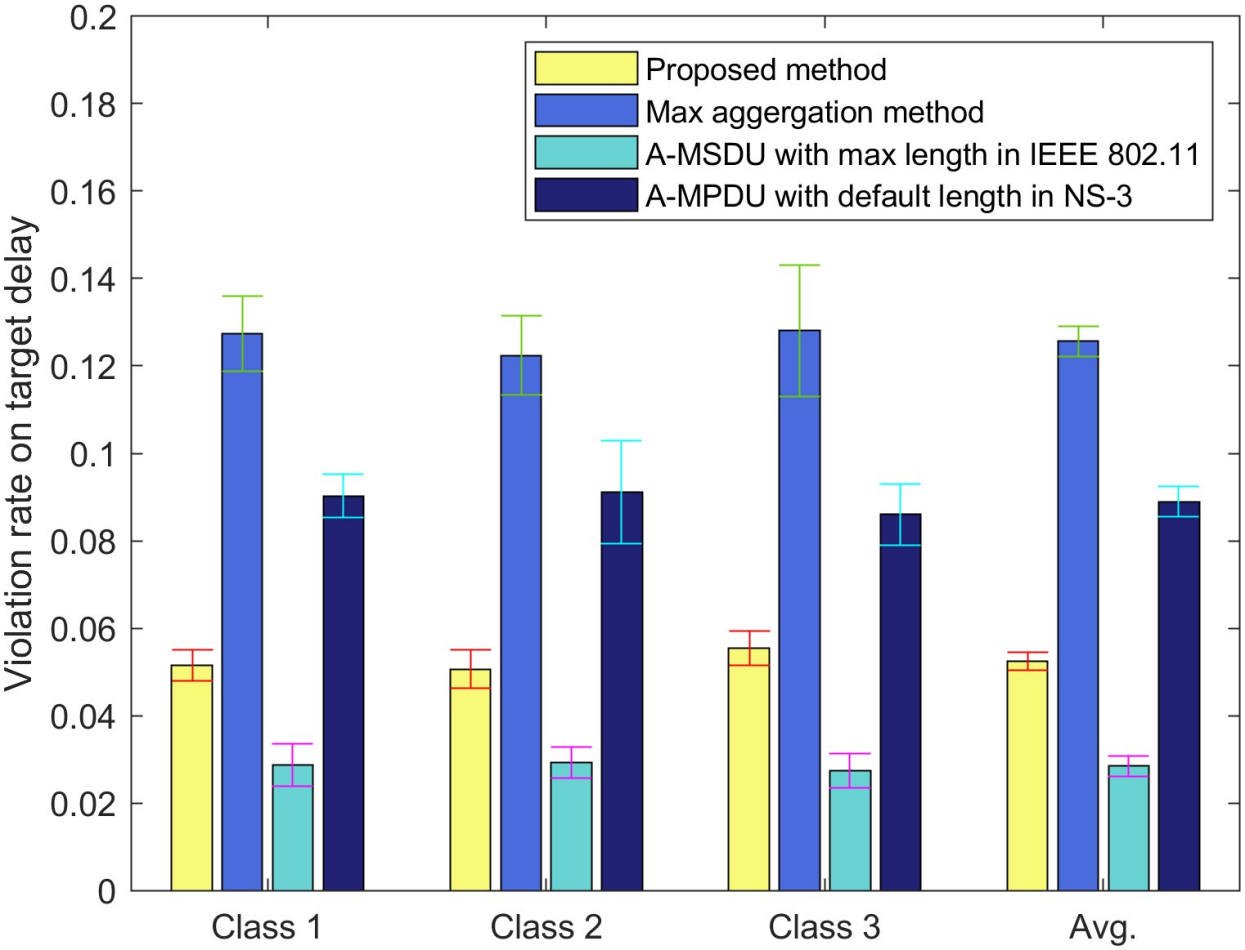
Comparison of the proposed method with other conventional methods for violation rate on the target delay of each class.

**Fig 9:**
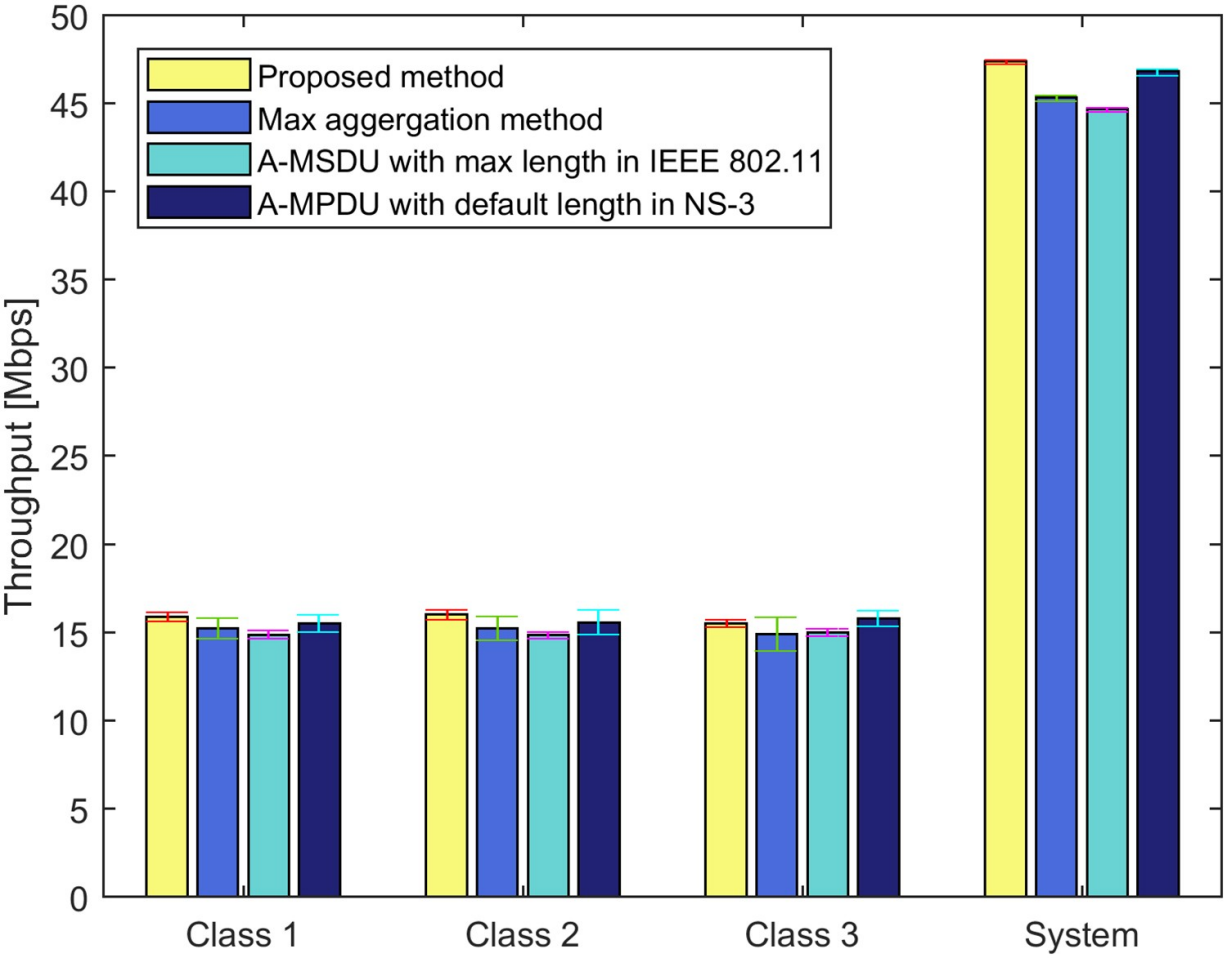
Comparison of the proposed method with other conventional methods for throughput of each class.

For Figs 8 and 9, we run NS-3 simulations with input parameter set III. The error bar indicates the standard deviation of the simulation results. In Figs 8 and 9, we compare our proposed method with the max aggregation method, A-MSDU using the value of the maximum length of A-MSDU in IEEE 802.11 standard (A-MSDU with the max length in IEEE 802.11) [1], and A-MPDU using the default value of the maximum length of A-MPDU in NS-3 (A-MPDU with the default length in NS-3) [18]. The target delays for classes *c* ∈ [1, 3] are same as 500 msec. From Eqs (1) to (14), we can obtain the same target number of aggregated MPDUs for classes *c* ∈ [1, 3] as 20, with the weight value *ω*_*i*_ = 0.5, *i* ∈ [1, 9] in the constraint (12). For A-MSDU with the max length in IEEE 802.11, the target number of aggregated MSDUs is 7 since the maximum length of A-MSDU is 11,454 bytes according to IEEE 802.11 standard [1]. For A-MPDU with the default length in NS-3, the target number of aggregated MPDUs is 42 since the default value of the maximum length of A-MPDU for the best effort traffic in NS-3 is 65,535 bytes [18]. In Figs 8 and 9, we set the maximum VHT PPDU duration as 10 sec which is longer than that of IEEE 802.11 standard to compare the proposed method with other conventional methods. Fig 8 shows the violation rate on the target delay of each class for each method. In Fig 8, the violation rate on the target delay of the proposed method is less than 0.1. The violation rate on the target delay of the proposed method is lower than those of the max aggregation method and A-MPDU with the default length in NS-3. For A-MSDU with the max length in IEEE 802.11 with input parameter set III, the violation rate on the target delay is the lowest in Fig 8, but the system throughput is the lowest in Fig 9 since the target number of aggregated frames is the smallest among four methods. Fig 9 shows the throughput of each class and the system throughput for each method. In Fig 9, A-MSDU with the max length in IEEE 802.11 yields the lowest system throughput, while our proposed method yields the highest system throughput among four methods.

## Conclusion

In this paper, we proposed a method that estimates an average delay of MPDUs for each queue and finds an optimal number of aggregated MPDUs for each queue to maximize the system throughput with satisfying the delay requirement of each queue in IEEE 802.11ac. We evaluated the performance of the proposed method through NS-3 simulations. The simulation results showed that the proposed method can estimate the average delay for each queue accurately. The simulation results also showed that the proposed method can obtain the violation rates on the target delays less than 0.1 because the proposed method uses the optimal number of aggregated MPDUs which is considered average delay. We also ran NS-3 simulations when the frame arrival process follows a Poisson distribution. The analytical results of the proposed method were close to simulation results when the frame arrival process follows a Poisson distribution. We compared the proposed method with other conventional methods through NS-3 simulations. The simulation results showed that the proposed method can yield higher system throughput than other conventional methods.
